# Abscisic-Acid-Regulated Responses to Alleviate Cadmium Toxicity in Plants

**DOI:** 10.3390/plants12051023

**Published:** 2023-02-23

**Authors:** Yuquan Zhao, Jiaqi Wang, Wei Huang, Dawei Zhang, Jinfeng Wu, Bao Li, Mei Li, Lili Liu, Mingli Yan

**Affiliations:** 1 School of Life and Health Sciences, Hunan University of Science and Technology, Xiangtan 411201, China; 2 Crop Research Institute, Hunan Academy of Agricultural Sciences, Changsha 410125, China; 3 Hunan Key Laboratory of Economic Crops Genetic Improvement and Integrated Utilization, Hunan University of Science and Technology, Xiangtan 411201, China; 4 Hunan Engineering and Technology Research Center of Hybrid Rapeseed, Hunan Academy of Agricultural Sciences, Changsha 410125, China

**Keywords:** exogenous ABA, Cd accumulation, abiotic stress, signal transduction, Cd-responsive gene

## Abstract

High levels of cadmium (Cd) in soil can cause crop yield reduction or death. Cadmium accumulation in crops affects human and animal health as it passes through the food chain. Therefore, a strategy is needed to enhance the tolerance of crops to this heavy metal or reduce its accumulation in crops. Abscisic acid (ABA) plays an active role in plants’ response to abiotic stress. The application of exogenous ABA can reduce Cd accumulation in shoots of some plants and enhance the tolerance of plants to Cd; therefore, ABA may have good application prospects. In this paper, we reviewed the synthesis and decomposition of ABA, ABA-mediated signal transduction, and ABA-mediated regulation of Cd-responsive genes in plants. We also introduced physiological mechanism underlying Cd tolerance because of ABA. Specifically, ABA affects metal ion uptake and transport by influencing transpiration and antioxidant systems, as well as by affecting the expression of metal transporter and metal chelator protein genes. This study may provide a reference for further research on the physiological mechanism of heavy metal tolerance in plants.

## 1. Introduction

Plants are immobile and fixed to soil; therefore, they may be subjected to adverse effects from abiotic stresses such as drought, heat, low temperature, nutrient, salt, and heavy metals [1]. These abiotic stresses greatly limit the distribution of plants, alter plant growth and development, and threaten global crop productivity [1,2].

Cadmium (Cd) is a highly toxic heavy metal that has no biological function and can persist in organisms for a long time (biological half-life: 10–30 years) [3,4]. Cadmium exposure can cause metabolic disorders in plant leaves, leading to excessive accumulation of reactive oxygen species (ROS) such as H_2_O_2_, further causing oxidative stress, cell death, and even plant death [5]. Excessive Cd uptake by plant roots leads to root browning, reduced root length and dry weight, altered root morphology, reduced root uptake capacity, cellular damage, and, ultimately, root death [5,6]. In addition, Cd accumulation in crops can endanger human health as Cd passes through the food chains [7,8]. Residents of Toyama Prefecture, Japan, have suffered from painful disease (Itai-itai disease) due to long-term consumption of Cd-contaminated rice (*Oryza sativa* L.) and water [9]. Studies have reported that long-term exposure to low doses of Cd can cause serious health effects [10]. Therefore, it is particularly important to eliminate Cd pollution in the environment, enhance heavy-metal tolerance in crops, and reduce Cd accumulation in crops.

Abscisic acid (ABA) is a sesquiterpene phytohormone that plays important roles in plant growth and development, e.g., in inducing dormancy in buds and seeds, causing stomatal closure, and promoting abscission of flowers and fruits [11,12,13,14,15]. Although ABA was discovered as early as the 1960s [11], the physiological effects of ABA have not been sufficiently studied until now. Seed dormancy is an adaptive mechanism in plants that senses external changes and rapidly enters a dormant state under unfavorable conditions. The precise response of seeds to environmental factors is mediated by different hormonal pathways, among which ABA is the main hormone that induces and maintains seed dormancy [16]. However, gibberellin (GA) plays an opposite role in this process, and when the effect of ABA is counteracted by GA, seeds will germinate at the proper time [16]. In addition, other growth regulators interact with ABA or GA, either synergistically or antagonistically, thus affect the state of the seeds [16]. The traditional view is that under unfavorable conditions such as drought stress, ABA produced by roots is transported to the leaves, where it causes stomatal closure. However, it has been reported that hydraulic signals can induce local production of ABA in leaves [12]. Subsequent researchers have even shown that ABA synthesis may also occur in the guard cells and in vascular tubes of nutrient organs [17]. Although researchers have different opinions about the site of ABA synthesis, they are in agreement about ABA causing stomatal closure in guard cells. Researchers also have different opinions regarding the abscission of leaves, flowers, and fruits. Initially, it was thought that abscisic acid caused the abscission of plant tissues [18]. Later, researchers suggested that possibly both ABA and ethylene play a role in abscission [19,20]. However, some researchers have also suggested that the role of ABA may be secondary and that it may be ethylene that plays a direct role [21]. Interestingly, a recent study suggests that ABA may cause leaf senescence in an ethylene-independent manner [22]. This may imply that ABA can cause leaf abscission in an ethylene-independent manner, a view that may indirectly support the second view.

In addition, ABA plays an active role as a natural “stress hormone” in plants’ response to abiotic stresses [2,12,23]. Studies have reported that the application of exogenous ABA has promising applications in reducing heavy-metal accumulation and enhancing heavy-metal tolerance in plants [2,23,24,25]. Under Cd stress, spraying ABA significantly reduced the cell death and accumulation of H_2_O_2_ and malondialdehyde in the root system of Pingyi sweet tea [5]; additionally, it reduced the leaf transpiration rate, Cd^2+^ influx in the root system, Cd content in plants, and transport of Cd from the root system to the shoots [5]. However, spraying fluridone (Flu), an inhibitor of ABA biosynthesis, exhibited the opposite effects [5]. Under Cd stress, ABA application reduced Cd^2+^ content in roots in Pingyi sweet tea (*Malus hupehensis* Rehd. var. pingyiensis Jiang) [7]. Meanwhile, ectopic expression of *MhNCED3* (a key gene for ABA synthesis) in transgenic *Arabidopsis thaliana* and apple (*Malus pumila Mill*) calli exhibited reduced expression of Cd^2+^-uptake-related genes (*NRAMP* and *IRT*) and reduced Cd^2+^ influx and Cd content [7]. Furthermore, inoculation with ABA-producing bacteria, namely, *Azospirillum brasilense* and *Bacillus subtilis*, reduced Cd levels in pakchoi (*Brassica chinensis* L.) (40–79% and 43–77%, respectively) in Cd-contaminated soil, reduced Cd-induced photosynthetic inhibition and oxidative damage, and increased the biomass of pakchoi (28–281% and 26–255% for the two bacteria, respectively) [26]. In addition, the alleviating effects of ABA to Cd stress have been reported in *Arabidopsis* [27], mung bean (*Vigna radiata* L.) [28], and poplar (*Populus euphratica*) [29]. The above studies suggest that the application of ABA to alleviate cadmium stress has good application prospects.

Here, we summarize the physiological mechanisms by which ABA enhances Cd tolerance in plants and ABA-mediated signal transduction pathways and regulation of Cd-responsive genes. This review may lay a foundation for the targeted enhancement of plants’ adaptation to the environment.

## 2. Synthesis and Catabolism of ABA in Plants

**Figure 1:**
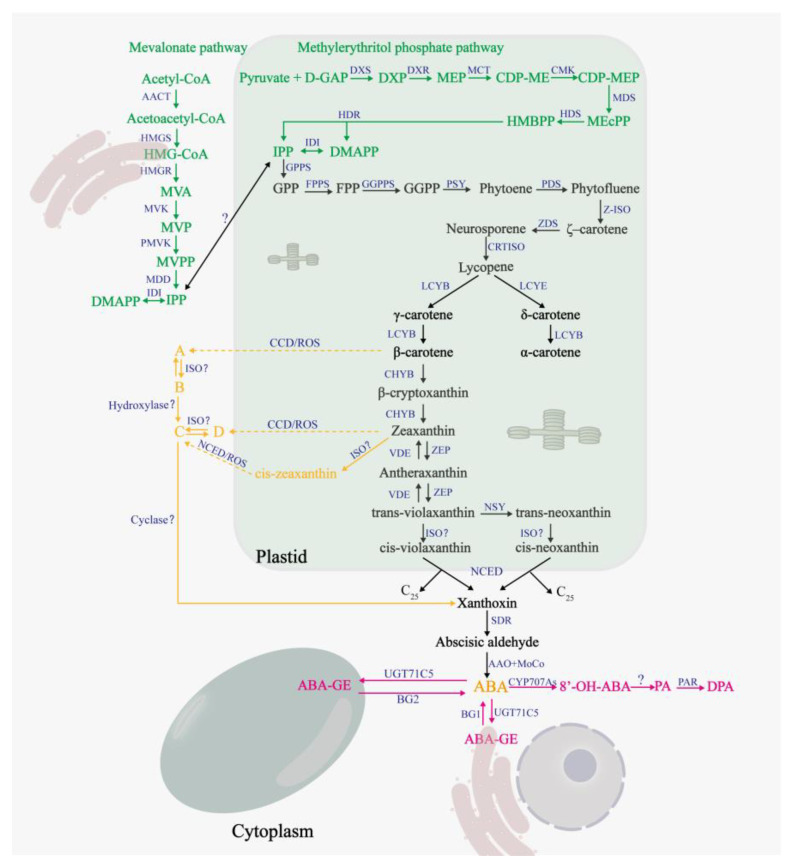
Biosynthesis and catabolic pathways of ABA. The mevalonate (MVA) pathway and methylerythritol phosphate (MEP) pathway are highlighted in green. The ABA synthesis pathway is shown in black, and the yellow part represents the zeaxanthin epoxidase (ZEP) -independent ABA synthesis pathway as proposed by Jia, Mi [34]. The ABA catabolic pathway and ABA coupling reaction are marked in red. Dashed arrows indicate multiple enzymatic steps. Enzymes are shown in blue, and unidentified enzymes are marked with question marks. Abbreviations of the enzymes or substrate that are not given in main text are as follows: (A) MVA and MEP pathway, acetyl-CoA C-acetyltransferase (AACT), HMG-CoA synthase (HMGS), HMG-CoA reductase (HMGR), mevalonate-5-phosphate (MVP), phosphomevalonate kinase (PMVK), MVPP decarboxylase (MDD), D-glyceraldehyde-3-phosphate (D-GAP), DXP synthase (DXS), DXP reductoisomerase (DXR), CDP-ME synthase (MCT), CDP-MEP kinase (CMK), MEcPP synthase (MDS), HMBPP synthase (HDS), HMBPP reductase (HDR). (B) ABA synthesis pathways. geranylgeranyl pyrophosphate (GPP) synthase (GPPS), farnesyl pyrophosphate (FPP) synthase (FPPS), GGPP synthase (GGPPS), phytoene synthase (PSY), phytoene desaturase (PDS), ζ-carotene isomerase (Z-ISO), carotenoid isomerase (CRTISO), lycopene β-cyclase (LCYB), lycopene ε-cyclase (LCYE), β-carotene hydroxylase (CHYB), violaxanthin de-epoxidase (VDE), neoxanthin synthase (NSY), isomerase (ISO), C25 metabolite (C25), abscisic aldehyde oxidase (AAO), molybdenum cofactor (MoCo), carotenoid cleavage dioxygenases (CCD). A, B, C, and D represent β-apo-11-carotenal, 9-cis-β-apo-11-carotenal, 9-cis-3-OH-β-apo-11-carotenal, and 3-OH-β-apo-11-carotenal, respectively. (C) ABA catabolic pathways. UGT71C5, a β-glucosyltransferase. β-glucosidases 1/2 (BG1/2). CYP707As, the gene encoding β-glucosyltransferase. PA reductase (PAR).

Abscisic acid plays an important role in normal growth and development as well as adaptive responses of plants to environmental stresses [30]. Humplik, et al. [31] proposed that, similar to other plant hormones, the effect (activation or inhibition) of ABA is determined by the dose and tissue sensitivity. For appropriate and accurate effect, ABA level is controlled through fine-tuning of de novo biosynthesis and catabolism [30,32]. Several studies have demonstrated that endogenous ABA content increased under Cd stress [7,33]. Similarly, the expressions of β-glucosyltransferase (an enzyme related to ABA uncoupling) and isoprenoid synthetase (an enzyme related to ABA synthesis pathway) were increased in *Tamarix hispida* under Cd stress [34]. Interestingly, under the same conditions, enzymes related to ABA synthesis pathway, such as ζ-carotene dehydrogenase (ZDS) and zeaxanthin epoxidase (ZEP), exhibited a downward trend [34]. These contradicting observations could be because of the varying sensitivity of the ABA-synthesis-related enzymes to Cd. The synthetic and catabolic pathways of ABA are shown in Figure 1. The synthesis of ABA requires five-carbon atom isoprene units, namely, isopentenyl diphosphate (IPP), and its isomer, namely, dimethylallyl diphosphate (DMAPP) [35,36,37]. IPP was synthesized through mevalonate (MVA) pathway and methylerythritol phosphate (MEP) pathway in plants [36,37,38,39].

In the IPP synthesis phase, both the MEP and MVA pathway can produce IPP through six consecutive steps [37,38,39]. IPP and DMAPP are interconverted by an IPP:DMAPP isomerase (IDI) [36,37].

In the early stages of carotenoid synthesis, IPP undergoes consecutive condensation reactions (one isoprene unit added at a time) to produce geranylgeranyl pyrophosphate, farnesyl pyrophosphate, and geranylgeranyl pyrophosphate (GGPP) sequentially [12]. The two molecules of GGPP are condensed head-to-head to form the colorless phytoene by the action of phytoene synthase [12,40]. Phytoene undergoes a four-step dehydrogenation process to produce the red trans-lycopene [12,35]. The trans-lycopene is cyclized by lycopene ε-cyclase or lycopene β-cyclase to introduce the ε- or β-rings, respectively [35,40]. A β-ring and an ε-ring are introduced to form α-carotene and its derivatives [35,40]; two β-rings are introduced to form β-carotene and its derivatives [35,40]. β-carotene undergoes hydroxylation catalyzed by β-carotene hydroxylase to produce β-cryptoxanthin, which is hydroxylated again to produce zeaxanthin [35]. Zeaxanthin epoxidase catalyzes the conversion of zeaxanthin to antheraxanthin, which in turn generates trans-violaxanthin [40,41]. Under strong light, trans-violaxanthin de-epoxidase can be reconstituted into zeaxanthin, via a reversible reaction called the xanthophyll cycle, which can provide good photoprotection to plants [41]. Trans-violet xanthin can be converted into cis-violaxanthin, and also into trans-neoxanthin and then into cis-neoxanthin [42]. In the plastid, cis-violet xanthin and cis-neoxanthin are cleaved to form xanthoxin and the C25 metabolite by the action of 9-cis-epoxycarotenoid dioxygenase (NCED) [43]. In the cytoplasm, short-chain dehydrogenase (SDR) catalyzes the production of abscisic aldehydes by xanthoxin [42]. Abscisic aldehydes are cleaved by abscisic aldehyde oxidase and molybdenum cofactor to produce ABA [42].

In addition, a ZEP-independent pathway for ABA synthesis starting from β-carotene and zeaxanthin was identified by Jia, et al. [44]. β-carotene and zeaxanthin are produced in the presence of carotenoid cleavage dioxygenases and ROS, respectively, to produce β-apo-11-carotenoid aldehyde and 3-OH-β-apo-11-carotenoid aldehyde [44]; the former produces 9-cis-β-apo-11-carotenoid aldehyde and 9-cis-3-OH-β-apo-11-carotenoid aldehyde, in turn, under the effect of isomerase and hydroxylase [44]. The latter can produce 9-cis-3-OH-β-apo-11-carotenal under the action of isomerase, and finally, 9-cis-3-OH-β-apo-11-carotenal generates ABA in the presence of cyclooxygenase [44].

Abscisic acid is catabolized by two main types of processes, namely, hydroxylation and conjugation [32,42]. Three different ABA hydroxylation pathways are observed depending on the position of the methyl group being oxidized (C-7′, C-8′, and C-9′) [30,42]. 8′-hydroxylation is the predominant hydroxylation pathway [32], whose product is 8′-OH-ABA [12]. Subsequently, 8′-OH-ABA is autonomously isomerized to phaseic acid (PA) [32], and PA is further converted into dihydro-phaseic acid (DPA) by PA reductase [45].

Basal levels of ABA play an important role in normal plant growth and development [46]. Under stress conditions, higher levels of ABA can help plants cope with the adverse external environment [47]. Therefore, the regulation of ABA levels in plants is very important, both under normal and stress conditions. As previously described, ABA levels are finely regulated by synthesis and catabolism; however, this is not sufficient. When the external environment changes drastically, the organism requires a more efficient way to respond than ABA synthesis and catabolism. The existence of the coupling cycle solves this problem. Under proper conditions, ABA and glucose are coupled into the inactive ABA-glucose ester (ABA-GE) [12]. ABA-glucose ester is the storage and transport mode of ABA [12], which is localized to the ER and vacuole [45]. Upon changes in external conditions, ABA-GE can be rapidly converted to ABA by BG1 (β-glucosidases 1) and BG2 (β-glucosidases 2), which are important for responding to adverse conditions [45].

## 3. Physiological Mechanisms Underlying Enhancement in Cd Tolerance in Plants by ABA

### 3.1. Regulation of Transpiration by ABA in Plants

In higher plants, the accumulation of heavy metals in leaves affects the function of stomata, which in turn affects transpiration. Studies have reported that transpiration rate is related to the xylem loading of Cd and is the main driver of Cd transport from roots to shoots [48,49,50,51]. The Cd content in Pingyi sweet tea roots increased with increasing leaf transpiration rate and decreased with decreasing transpiration rate [5]. Transpiration inhibitors such as paraffin and CaCl_2_ could reduce Cd content in tobacco (*Nicotiana tabacum* L.) leaves, and the reduction in Cd content was linearly correlated with the transpiration rate of tobacco leaves [48]. Consistent with the above findings, previous related studies in maize (*Zea mays*), mustard (*Brassica juncea* L.), rice, and wheat (*Triticum aestivum*) reported that higher transpiration rates were associated with higher Cd levels in shoots [52].

Stomata regulate the parallel diffusion pathways of water and CO_2_ between leaves and atmosphere, thus playing a regulatory role in transpiration and photosynthesis [53]. Studies have reported that ABA regulates transpiration by modulating stomatal aperture [28,50,52,54]. Abscisic acid treatment reduced transpiration rate by 72% and 64% in Habataki and Sasanishiki (two rice cultivars), respectively, resulting in the reduction in Cd accumulation in rice shoots [55]. After spraying ABA, the transpiration rate of Pingyi sweet tea leaves and Cd^2+^ uptake and accumulation in the root system reduced, mitigating damage to the root system due to Cd [5]. Under Cd stress, endogenous ABA levels of non-hyperaccumulation ecotype *Sedum alfredii* increased, which reduced the size and density of stomata in the leaves and also reduced the transport of Cd^2+^ to the shoots [52]. In contrast, under Cd stress, endogenous ABA contents in hyperaccumulation ecotype *S. alfredii* were maintained only at low levels, and they could not limit transpiration rate, thus exhibiting higher Cd accumulation [52]. The above findings suggested that the reduction in Cd content caused by ABA is closely related to the inhibition of transpiration.

### 3.2. Regulation of Metal Ion Transport by ABA in Plants

In the inter-root environment of rice, heavy metal ions are transported to rice roots by specific transporter proteins through the plasma membrane and then to the xylem or phloem via the plastid extracellular pathway or symplast pathway [51,56]; further, they are transported to various organs in shoots [51,56]. However, Cd does not have its own transporter, and it enters the plant body through the transporter of essential elements (e.g., Zn, Fe, and Ca) [4,57]. A study reported that Cd can enter the rice root system through OsIRT1 [57], and similarly, *Arabidopsis irt1* mutants have lower Cd levels than wild-type plants [58].

Abscisic acid reduced the transcript levels of IRT1 in cucumber (*Cucumis sativus* L.) and *Arabidopsis* roots [59]. Similarly, exogenous ABA could significantly reduce the Cd levels in the shoots of wild-type *Arabidopsis* [27]. However, its effect on *Arabidopsis irt1* mutants was not significant, and the addition of iron-regulated transporter 1 (IRT1) inhibitors eliminated the difference between Cd levels in shoots and roots in wild-type *Arabidopsis* with and without the addition of ABA [27]. Fan, et al. [60] reported that the application of 0.5 µM ABA under 10 µM Cd stress led to reduction in *IRT1* transcript levels by 90% in *Arabidopsis* roots; however, in ABA-insensitive double mutant snrk2.2/2.3, the repression of *IRT1* by ABA was not as pronounced as in wild-type *Arabidopsis* [60]. Consistent with the above findings, inoculation of ABA-producing bacteria in soil under Cd stress significantly downregulated the expression of root *IRT1*, which in turn inhibited Cd uptake by *Arabidopsis* [61]. However, inoculation of ABA-producing bacteria had little effect on Cd levels in *Arabidopsis irt1* knockout mutant [61]. These results suggested that the decrease in plant Cd levels induced by ABA application is mainly achieved through the regulation of *IRT1*.

Notably, a recent study by reported that the regulation of Cd accumulation in plants by ABA was related to the concentration of Fe^2+^ in the external environment [62]. Under the condition of sufficient Fe^2+^, ABA significantly inhibited the IRT1 expression and reduced Cd accumulation [62]. Under Fe^2+^-deficient conditions, ABA may regulate Cd accumulation by promoting the redirection of Fe in the ectoplasm [62]. This suggested that ABA-mediated regulation of Cd accumulation is a complex process.

Heavy metal ATPase 3 (HMA3) is a protein localized on the vesicle membrane and is responsible for the transport of Cd and Zn into the vesicles [63]. In rice, OsHMA3 is responsible for the segregation of Cd into the root vesicles [64]. Plants possessing nonfunctional *OsHMA3* exhibited increased transport of Cd from the roots to shoots [4]. Conversely, overexpression of *OsHMA3* enhanced Cd segregation and thus reduced Cd transport from the roots to shoots [4]. Unlike HMA3, the function of its homologous protein, HMA2, is not clear enough. Heavy metal ATPase 2 and ATPase 4 (HMA2 and HMA4) are present on the plasma membrane of thin-walled cells of vascular bundles and mediate the transport of Cd and Zn from the roots to shoots [4,63,65]. The functional deficiency of *HMA2* and *HMA4* in *Arabidopsis* resulted in the almost complete loss of Cd transport from the roots to shoots [65]. Rice *HMA2* mutants exhibited reduced Cd and Zn translocation rates from roots to shoots [66,67]. Similarly, knockout of HMA2 lowered Cd and Zn content in in the reproductive organs of rice [4]. In addition, Ectopic expression of *BrpHMA2* enhanced Cd accumulation in transgenic *Arabidopsis* and yeast [68]. Contradictorily, OsHMA2 overexpression plants showed reduced seed Cd concentration [67]. Therefore, further studies are necessary. In a study related to Cd accumulation in *S. alfredii*, researchers indicated that ABA increased Cd resistance and Cd transport from roots to shoots in *S. alfredii* through the induction of *HMA3* and *HMA4* transcripts [69]. However, *HMA2* expression was negatively correlated with endogenous ABA content [69], which implied that ABA may inhibit *HMA2* expression. Similarly, ectopic expression of *MhNCED3* in *Arabidopsis* reduced *AtHMA2* expression [7]. These results suggested that ABA may mediate the expression of some genes of the HMA family to affect Cd transport and accumulation.

Natural resistance-associated macrophage protein (NRAMP) family genes are involved in transmembrane transport of divalent heavy metal ions (including Cd^2+^) and play an important role in response to heavy metal stress [70,71]. Under Cd stress, soybean *NRAMP* genes were significantly upregulated [71]. Similarly, the expression of potato *NRAMP* genes (NRAMP1–5) significantly increased under Cd stress [70]. In rice, OsNRAMP5 was thought as the main Cd transporter [4,64]. A recent study reported that high expression of *OsNRAMP5* reduced Cd accumulation in rice seeds [64]. Natural resistance-associated macrophage protein family genes have been reported to be regulated mainly by phytohormones and transcription factors under abiotic stress [70]. A study indicated that increased ABA synthesis suppresses *NRAMPs* expression [7]. However, Zhou and Yang [72] reported that ABA downregulated the expression of *OsNRAMP1* but upregulated *OsNRAMP2* and *OsNRAMP3*. No evidence is available regarding the regulation of *NRAMP5* by ABA. Therefore, further research is necessary.

The aforementioned metal transporters are often involved in the transport of essential elements. For example, IRT1 is involved in Fe^2+^ transport [4]; OsHMA2 is required for Zn transport, and OsNRAMP5 is the main protein for Mn uptake and transport [4,64]. However, knockdown or overexpression of these genes often affect crop yield, which limits their application in breeding. Notably, overexpression of *OsHMA3* did not affect rice yield [64]. Therefore, *HMA3* has exhibited potential for the application in crop breeding.

### 3.3. Regulation of Metal Ion Sequestration by ABA in Plants

Despite entering the root cells, most Cd still cannot reach the shoots [4]. Metal ions can be chelated by reduced glutathione (GSH), phytochelatins (PCs), and nicotianamine (NA) [69,73,74]. These three share a common precursor: cysteine (Cys) [73,75]. Metal ions are segregated into vesicles via transport proteins after chelation, effectively ensuring that free metals are at low levels in the cytosol [73]. *Arabidopsis* synthesizes GSH via γ-glutamylcysteine synthase 1 (GSH1) and glutathione synthase 2 (GSH2) [23]. In plants, algae, and some fungi or worms [76,77], phytochelatin synthase (PCS) may catalyzes glutathione tripeptide γ-Glu-Cys-Gly (GSH) to synthesize PCs ((γ-Glu-Cys)n-Gly, *n* = 2–11) [74,78]. Studies show that Cd, As, and Pb in the cytoplasm can be coupled by GSH or PCs and then segregated into vesicles to alleviate the toxicity of heavy metals to cells [23]. A study reported that the formation of PC–Cd complexes is the main mechanism of Cd detoxification in *Arabidopsis* [65]. Consistent with these results, the accumulation of PCs in the root system is responsible for the higher Cd tolerance in wheat and higher Cu, Zn, and Cd tolerance in aquatic plants [73].

Studies have reported that the activity of PCS is the main cause of sequestration of heavy metals, such as Cd, As, and Hg, in plants [76,79,80,81]. Abscisic acid can alleviate metal stress by regulating the transcript level of *PCS*. In ramie (*Boehmeria nivea*), Cd and ABA could significantly induce *BnPCS1* [82]. In grey poplar (*Populus × canescens*), the application of exogenous ABA increased the transcript levels of *PCS* [59]. Consistent with these results, Cd or ABA treatment increased the transcript levels of *StPCS1* and PCS activity in potato (*Solanum tuberosum*) roots, whereas the addition of Flu decreased the transcript levels of *StPCS1* and PCS activity [83]. These results may indicate that ABA plays an important role in the metal ion chelation process.

### 3.4. Regulation of the Antioxidant System in Plants by ABA

Under normal physiological conditions, a balance exists between the production and clearance of ROS in all intercellular compartments. However, this balance may be disturbed by some adverse environmental factors. One of the main consequences of the action of heavy metals, including Cd, is enhanced ROS production, which leads to damage to membranes, nucleic acids, and proteins and impairment of normal cellular functions [84]. In turn, plants mitigate the damage caused by ROS through antioxidant systems [85]. For example, in land cotton, the expression of superoxide dismutase (SOD), ascorbate peroxidase (APX), and GSH is increased in response to Cd stress [86].

In poplar, ABA significantly increased the activities of antioxidant enzymes such as SOD, catalase (CAT), and APX, which scavenged Cd-induced ROS [29]. Similarly, ABA pretreatment alleviated Cd toxicity in roots by modulating the antioxidant defense system in mung bean seedling [87]. Exogenous ABA significantly increased the activities of antioxidant enzymes (SOD, CAT, and APX), which in turn scavenged excess ROS and protected cell membranes from oxidative damage by ROS [88]. Meanwhile, in purple flowering stalk (*Brassica campestris* L. ssp. *chinensis* var. *purpurea* Hort.), ABA alleviated the toxicity of Cd by activating the antioxidant enzyme system to reduce ROS [89]. In addition, exogenous ABA addition can lead to increases in non-enzymatic antioxidants such as ascorbic acid, GSH, carotenoids, and α-tocopherol [28]. Among them, GSH is a major antioxidant that scavenges excess ROS, maintains cellular redox homeostasis, and regulates protein function [85]; therefore, it plays an important role in plant survival under adverse conditions [85]. In addition, GSH can induce the expression of many downstream Cd-tolerance-related genes through the ABA signaling pathway [90]. Interestingly, ABA pretreatment can restore the level of GSH reduced by Cd stress and indirectly regulate oxidative stress caused by ROS accumulation under Cd stress [87]. These results suggested that ABA can alleviate Cd stress by regulating the antioxidant system in plants.

### 3.5. Other Regulatory Effects of ABA

In *Arabidopsis*, under the effect of proton pumps, more NO_3_^−^ and Cd^2+^ accumulate in the vesicles of root cells of *Arabidopsis*, thus reducing the toxicity of Cd^2+^ to the cells [91]. Nitrate transporter 1.5 (NRT 1.5) is a long-distance transporter of NO_3_^−^ [91]. *Arabidopsis* NRT 1.5 is expressed mainly in the mid-column sheath cells in roots and is involved in loading NO_3_^−^ into the xylem [91,92]. By inhibiting NRT 1.5 expression, ABA affects NO_3_^−^ partitioning in the root system, allowing more NO_3_^−^ to accumulate in the root system and thereby increasing Cd tolerance in plants [91,92].

## 4. ABA-Mediated Signal Transduction

The core components of the ABA signaling pathway are composed of the ABA receptors PYR/PYL/RCAR, the phosphatases PP2Cs, and the protein kinase SnRK2. PYR1 and PYL1–13 are ABA receptors, and RCAR1–14 are regulatory components of the ABA receptors [93]. Phosphatases PP2Cs are a group of monomeric Ser/Thr phosphatases whose activity is dependent on Mg^2+^ and Mn^2+^ [94]. *Arabidopsis* PP2Cs can be divided into 10 groups (A–J), and most of the A-type PP2Cs (including ABI1, ABI2, and HAB1) are involved in ABA signaling [95]. The *Arabidopsis* genome contains 38 SnRKs divided into three groups: SnRK1 (1.1–1.3), SnRK2 (2.1–2.10), and SnRK3 (3.1–3.25) [95]. The SnRK2 families are a group of ABA-activated protein kinases involved in abiotic stress signaling [95]. The SnRK2 family of 10 members can be further divided into three groups: I, II, and III. Group I (SnRK2.1, SnRK2.4, SnRK2.5, and SnRK2.9) does not respond to ABA [95]; group II (SnRK2.7 and SnRK2.8) is weakly activated by ABA [95]; and group III (SnRK2.2, SnRK2.3, and SnRK2.6) is strongly activated by ABA [95]. Group III was identified as the main positive regulator of ABA signaling [95]. Among them, SnRK2.6 is closely associated with plant stomatal opening and closing, and ABA-induced stomatal closure is disrupted in *Arabidopsis* SnRK2.6 deletion mutants [95]. A-type PP2Cs are efficient inhibitors of SnRK2 when ABA content is at basal levels [93], when SnRK2 kinase is inactive, and when transcription factors mediating the expression of ABA-responsive genes cannot be activated. Under stress conditions, ABA concentration increases because of increased ABA biosynthesis and reduced degradation or release of the conjugated form ABA-GE [96]. It induces PYR/PYL/RCAR binding and inhibits PP2Cs, which reverts the inhibition of SnRK2 by PP2Cs. Therefore, SnRK2 can autophosphorylate and activate downstream effectors, such as ion channels and transcription factors [95], thereby initiating the transcription of ABA-responsive genes [97].

**Figure 2:**
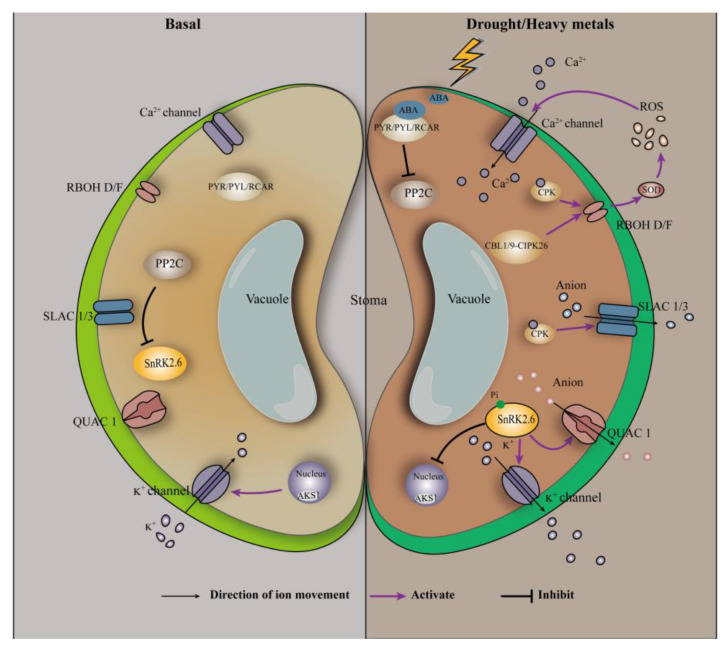
ABA-involved signal transduction in the guard cells. Adapted from Hauser, et al. [102].

Drought or heavy metal stress causes stomatal closure of plant cells, which is achieved through ABA-mediated signal transduction (Figure 2). The guard cells regulate stomatal pore apertures by integrating endogenous hormonal stimuli and environmental signals [98]. The stress hormone ABA and environmental signals (e.g., CO_2_) activate complex signaling pathways in guard cells mediated by kinase/phosphatase, secondary messengers, and ion channel regulation [98]. ABA has been reported to affect stomatal aperture size through both Ca^2+^-dependent and -independent pathways [45]. In the Ca^2+^-dependent pathway, stomatal closure requires an increase in intracellular Ca^2+^ concentration in the guard cells. Abscisic acid induces PYR/PYL/RCAR to derepress PP2Cs on the protein kinase SnRK2.6/OST1 [96]; activated SnRK2.6/OST1 activates the plasma-membrane-bound respiratory burst oxidase homolog (RBOH), which subsequently catalyzes ROS production via extracellular SOD [96]. In addition, Ca^2+^-bound calcineurin B subunit-like proteins (CBLs) interact with and regulate the activity of CBLs-interacting protein kinases [96]. The CBL1/CBL9-CIPK26 complex interacts with the N-terminal of RBOH F protein (RBOHF) and phosphorylates RBOHF, leading to an increase in RBOHF-mediated ROS production [96]. ROS, particularly H_2_O_2_, can further promote the opening of Ca^2+^ channels, thus increasing the Ca^2+^ concentration in the guard cells [45,99]. Changes in Ca^2+^ concentration are sensed by several Ca^2+^ sensors, including calcium-dependent protein kinase 3/4/6/10/11, which may phosphorylate and activate slow-type anion efflux channels including slow anion channel associated 1 (SLAC1) and slow anion channel 3 [45,99,100]; this ultimately leads to stomatal closure. In the Ca^2+^-independent pathway, activated SnRK2.6/OST1 directly binds and phosphorylates SLAC1 and the quick activating anion channel (QUAC1) [96,101]. Through activated SLAC1 and QUAC1 channels, the defense of the cell’s fast anion efflux depolarizes the plasma membrane, which activates K^+^ efflux channels, thereby driving K^+^ efflux and solute release from the guard cell [101]; this ultimately leads to stomatal closure. By comparing the Ca^2+^-dependent and -independent pathway, it is not difficult to find that the protein kinase SnRK2.6 does play an important role in the stomatal closure process, which is consistent with the previous statement. This is because both Ca^2+^-dependent and -independent pathways require SnRK2.6 to activate the downstream effector, which in turn triggers the next step of the reaction.

## 5. Regulation of Cd-Responsive Genes in Plants by ABA

**Figure 3:**
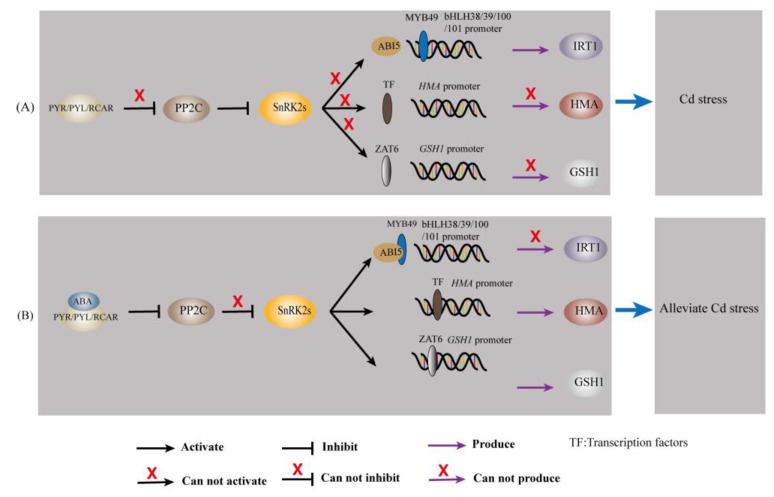
ABA regulates Cd-responsive genes in plants. (**A**) The transcription of *IRT1*, *HMA*, and *GSH1* under Cd stress. (**B**) The transcription of *IRT1*, *HMA*, and *GSH1* when ABA was involved under Cd stress.

Abscisic acid transmits messages through a signal transduction pathway that ultimately translates the initial stress signal into changes in gene expression [97]. When environmental conditions are harsher (e.g., Cd stress), ABA levels increase and subsequently induce PYR/PYL/RCAR binding and repression of PP2Cs, which deregulates SnRK2 inhibition [95,97]. Thus, SnRK2 can autophosphorylate and activate downstream effectors such as ion channels and transcription factors (ZAT6, bZIP (including ABI5), MYB, etc.) [95,97]. Transcription factors further bind to cis-acting elements (e.g., ABRE) in the target gene promoter, thereby controlling the expression of the target gene [97]. The regulatory role of ABA on Cd-responsive genes is given in Table 1 and Figure 3.

Iron-regulated transporter 1, a member of the ZIP family, has broad specificity for divalent metal ions and is the main mode of Fe and Cd uptake by plants [8,58]. Abscisic acid upregulates the expression of the bZIP transcription factor ABI5 [23,103]. This causes its protein product to bind to the R2R3-MYB transcription factor MYB49, which prevents MYB49 from binding to the promoter of the downstream gene bHLH38/39/100/101 [23,103]; thereby, IRT1 expression is inhibited, and Cd accumulation is reduced [23,103].

The HMA gene family mainly encodes P1b-type ATPases, which use the energy released from ATP hydrolysis to drive the transmembrane transport of heavy metal ions [104]. Li, et al. [105] applied genomic approaches and reported that *HMA1* and *HMA4* contain ABA-responsive elements (ABREs and AREs). In addition, a study indicated that the promoter regions of all nine *HMA* genes identified in *Medicago truncatula* contained ABA response elements [106]. These findings suggested that ABA may influence Cd accumulation in plants and plants’ tolerance to Cd by regulating the expression of *HMA*.

Chen, et al. [107] used forward genetics to identify a Cd-resistance *Arabidopsis* mutant, namely, *xcd2-D*, with a mutant gene encoding the transcription factor ZAT6. Overexpression of *ZAT6* significantly enhanced Cd tolerance in *Arabidopsis* plants, whereas loss of function of *ZAT6* increased the sensitivity of plants to Cd [107]. Further studies revealed that *ZAT6* positively regulates the transcription of *GSH1*, *GSH2*, *PCS1*, and *PCS2* and can specifically bind to the promoter of *GSH1* in vivo [107]. A study indicated that exogenous ABA treatment upregulated the expression of *ZAT6* [108]. Abscisic acid may regulate the expression of *GSH1*, *GSH2*, *PCS1*, and *PCS2* by regulating *ZAT6* and further affect Cd accumulation in plants and plants’ tolerance to Cd. These findings suggested that ABA may affect metal ion chelation through upregulation of *ZAT6*.

*ThUGT* is the gene encoding β-glucosyltransferase in *T. hispida*. Under Cd stress, *ThUGT* enhances the resistance of *T. hispida* to Cd by regulating ROS production and inhibiting Cd uptake [34].

**Table 1:** Regulation of Cd-responsive genes in plants by ABA.

Species	Genes	Regulating Effects	References
*Arabidopsis thaliana*	*ZAT6*	ABA could upregulate *ZAT6*, and the latter positively regulates Cd accumulation and tolerance in *A. thaliana*.	[107,108]
	*IRT1*	Compared with the control group, the expression of IRT1 in the roots of *A. thaliana* treated with CdCl_2_ and ABA decreased by approximately 90%.	[60]
	*ABI5*	ABA-induced ABI5 interacts with Cd-induced MYB49 to reduce Cd uptake and accumulation.	[103]
*Brassica juncea* L. *Czern. et Coss.*	*BJCDR15* and *TGA3*	ABA upregulated *BJCDR15* and *TGA3* (particularly the latter), and *BJCDR15* overexpression in *A. thaliana* and tobacco increased Cd accumulation. The *A. thaliana* mutant *TGA3-2* had a high Cd content in its roots, and Cd transport was blocked.	[109]
*Boehmeria nivea*	*BnPCS1*	Cd and ABA significantly induced *BnPCS1* expression. Plants overexpressing *BnPCS1* accumulated more Cd.	[82]
	*BnbZIP3*	ABA treatment could induce *BnbZIP3* expression. Overexpression of *BnbZIP3* alleviated Cd stress.	[110]
*Hevea brasiliensis*	*HbMT2a*	ABA could upregulate *HbMT2a*. Overexpression of *MT2s* could increase plants’ resistance to Cd.	[111]
*Juglans regia* L.	*JrVHAG1*	CdCl_2_ and ABA significantly upregulated *JrVHAG1*. Overexpression of *JrVHAG1* improved the growth of *A. thaliana* under ABA and/or CdCl_2_ treatment and increased the activity of antioxidant enzymes.	[112]
*Solanum lycopersicum*	*TCMP-1*	*TCMP-1* responded to Cd and ABA, and TCMP-1 interacted with heavy-metals-associated HIPP26 in tomatoes. Cd accumulation was lower in *A. thaliana* overexpressing *TCMP-1*.	[113]
*Oryza sativa*	*OsSMP1*	ABA could upregulate *OsSMP1*, and *OsSMP1* overexpression could improve the tolerance of rice to CdCl_2_ and CuSO_4_.	[114]
*Poa Pratensis*	*Dof*, *MADS25*, *BCR-BPC*, etc.	Dof, MADS25, BCR-BPC, B3, bZIP23, and MYB30 may be the central transcription factors under Cd stress. Hormonal signals, including ABA, interact with them to regulate the expression of multiple genes related to cell wall membrane stability and Cd tolerance.	[115]
*Sedum alfredii*	*HsfA4c*	*Hsfs* plays an important role in stress resistance. Treatment with ABA or Cd enhanced the expression of *HsfA4c*.	[69]
	*NAS*	*NAS* encodes nicotianamine, which is involved in the long-distance transport of metals. The expression of *NAS* was positively correlated with endogenous ABA content.	
	*HMA3*	*HMA3* encodes a metal transporter whose expression in the shoots correlated with endogenous ABA content.	
	*CAD*	*CAD* encodes a protein associated with cell wall synthesis, and its expression positively correlated with endogenous ABA content.	
	*HMA4*	HMA4 is a transporter that enhances Cd tolerance and promotes transfer of Cd to the shoots. Under the co-treatment of ABA and Cd, the expression of *HMA4* was higher and positively correlated with endogenous ABA content and Cd accumulation.	
*Saccharum*	*ScGluD2*	*ScGluD2* is involved in responding to heavy metal stress in sugarcane, and ABA plays a role in *ScGluD2* activation induced by CdCl_2_.	[116]
*Triticum aestivum*	*WRAB15* and *WRAB18*	*WRAB15* and *WRAB18* were regulated by ABA and induced by Cd, and their expression levels in wheat seedling leaves positively correlated with seedlings’ resistance to Cd.	[117]
*Zea mays*	*GSH1*	GSH treatment restored plant growth, root cell viability, photosynthetic capacity, REDOX balance, and cell ultrastructure. Meanwhile, Cd-tolerance-related genes were strongly upregulated. Under Cd stress, ABA content significantly decreased after GSH application, except in leaves.	[90]
*Tamarix hispida*	*ThUGT*	*ThUGT* is a gene of the ABA signaling pathway, and overexpression of *ThUGT* could reduce Cd accumulation.	[34]

## 6. Conclusions

Endogenous ABA levels in plants tend to be elevated when subjected to abiotic stresses. This is caused by increased ABA synthesis and reduced catabolism and conjugation reactions. When an organism does not require much ABA, extra ABA can be broken down into PA and DPA or transformed into ABA-GE (the inactive conjugated form of ABA). When ABA is required in some tissues, ABA-GE can be rapidly converted into active ABA to help the plants cope with abiotic stresses.

Many studies have reported that ABA plays positive roles in enhancing the tolerance of plants to Cd. The physiological mechanisms underlying the enhancement of Cd tolerance in plants by ABA can be summarized as follows: (1) attenuating transpiration, thereby reducing Cd uptake and transport of Cd from the roots to shoots; (2) inhibiting the expression of metal transporter proteins genes to reduce Cd uptake and transport; (3) increasing the expression of phytochelatin synthase gene, which in turn enhances Cd chelation; and (4) increasing antioxidant enzyme activity or nonenzymatic antioxidant content to improve tolerance of plants to Cd.

In fact, the physiological changes in plants under stress are closely related to the transduction of stress signals and changes in the expression of stress-responsive genes. Under Cd stress, ABA affects stomatal aperture size through both Ca^2+^-dependent and -independent pathways, thereby affecting transpiration and reducing Cd uptake. Moreover, ABA regulates the genes encoding metal transport proteins, such as *IRT1* and *HMA*, thereby reducing metal ion uptake and transport. In addition, ABA can promote the expression of *GSH1*, which in turn increases the chelation of metal ions and alleviates the toxicity of Cd^2+^ to cells.

Studies have shown that ABA plays an important role both in the normal growth and development of plants as well as in response to adversity. It is necessary to reveal the mechanism of ABA’s action at the molecular level. Therefore, future research work can be carried out in the following aspects.

1. Search for genes that respond to both ABA and stress. Search for transcription factors upstream of genes, and improve the gene network involved in the regulation of ABA. This can be combined with transgenic technology (gene knockout and overexpression) to fundamentally breed stress-resistant crops, which is of great importance for molecular breeding.
2. Identify unknown components of the ABA signaling pathway, improve the ABA signaling network, and understand the crosstalk between the ABA signaling pathway and other signaling pathways (e.g., MAPK). This will facilitate the understanding of how the adverse external environment causes the plant to develop a resistance response.
3. Understand the mechanism of crosstalk between ABA and other hormones. This will be extremely helpful in understanding plant physiology and developing integrated resistance strategies. Like genes, hormones often do not work in isolation.
4. Uncover new stress receptors. This is necessary to elucidate the mechanism of stress response.
5. Explore whether ABA has a dosage effect and the differences in sensitivity among plant tissues. This will hopefully improve stress resistance without affecting yield.

## Data Availability

Not applicable.
